# Genomic and Phylogenetic Analysis of *Bacillus cereus* Biovar *anthracis* Isolated from Archival Bone Samples Reveals Earlier Natural History of the Pathogen

**DOI:** 10.3390/pathogens12081065

**Published:** 2023-08-20

**Authors:** Michael H. Norris, Diansy Zincke, David J. Daegling, John Krigbaum, W. Scott McGraw, Alexander Kirpich, Ted L. Hadfield, Jason K. Blackburn

**Affiliations:** 1 Spatial Epidemiology & Ecology Research Laboratory, Department of Geography, University of Florida, Gainesville, FL 32611, USA; mhnorris@ufl.edu (M.H.N.); dzincke@ufl.edu (D.Z.); thadfield@ufl.edu (T.L.H.); 2 Emerging Pathogens Institute, University of Florida, Gainesville, FL 32611, USA; 3 Department of Anthropology, University of Florida, Gainesville, FL 32611, USA; daegling@ufl.edu (D.J.D.); krigbaum@ufl.edu (J.K.); 4 Department of Anthropology, Ohio State University, Columbus, OH 43210, USA; mcgraw.43@osu.edu; 5 Department of Population Health Sciences, Georgia State University, Atlanta, GA 30302, USA; akirpich@gsu.edu

**Keywords:** anthrax, emerging infections, Bcbva, *Bacillus*, toxin, phylogenomics

## Abstract

(1) Background: *Bacillus cereus* biovar *anthracis* (Bcbva) was the causative agent of an anthrax-like fatal disease among wild chimpanzees in 2001 in Côte d’Ivoire. Before this, there had not been any description of an anthrax-like disease caused by typically avirulent *Bacillus cereus.* Genetic analysis found that *B. cereus* had acquired two anthrax-like plasmids, one a pXO1-like toxin producing plasmid and the other a pXO2-like plasmid encoding capsule. Bcbva caused animal fatalities in Cameroon, Democratic Republic of Congo, and the Central African Republic between 2004 and 2012. (2) Methods: The pathogen had acquired plasmids in the wild and that was discovered as the cause of widespread animal fatalities in the early 2000s. Primate bones had been shipped out of the endemic zone for anthropological studies prior to the realized danger of contamination with Bcbva. Spores were isolated from the bone fragments and positively identified as Bcbva. Strains were characterized by classical microbiological methods and qPCR. Four new Bcbva isolates were whole-genome sequenced. Chromosomal and plasmid phylogenomic analysis was performed to provide temporal and spatial context to these new strains and previously sequenced Bcbva. Tau and principal component analyses were utilized to identify genetic and spatial case patterns in the Taï National Park anthrax zone. (3) Results: Preliminary studies positively identified Bcbva presence in several archival bone fragments. The animals in question died between 1994 and 2010. Previously, the earliest archival strains of Bcbva were identified in 1996. Though the pathogen has a homogeneous genome, spatial analyses of a subset of mappable isolates from Taï National Park revealed strains found closer together were generally more similar, with strains from chimpanzees and duikers having the widest distribution. Ancestral strains were located mostly in the west of the park and had lower spatial clustering compared to more recent isolates, indicating a local increase in genetic diversity of Bcbva in the park over space and time. Global clustering analysis indicates patterns of genetic diversity and distance are shared between the ancestral and more recently isolated type strains. (4) Conclusions: Our strains have the potential to unveil historical genomic information not available elsewhere. This information sheds light on the evolution and emergence of a dangerous anthrax-causing pathogen.

## 1. Introduction

*Bacillus cereus* are generally thought of as nonpathogenic saprophytic Gram-positive spore formers [1]. Occasionally, *B. cereus* is linked to food borne illness [2]. The closely related bacterium, *Bacillus anthracis*, is associated with the severe disease anthrax. *B. anthracis* uses two virulence plasmids, pXO1 and pXO2, to cause anthrax. Without the anthrax toxin-encoding plasmid pXO1, *B. anthracis* retain a marginal level of pathogenicity but have been used as vaccines [3,4]. Pasteur-like attenuated vaccines are based on strains that do not have the pXO1 plasmid. These vaccines have fallen out of favor due to their retention of pathogenicity. Pasteur-like vaccines maintain plasmid pXO2, encoding the poly-g-D-glutamic acid capsule that allows vegetative cell survival in animals. Sterne-like vaccines lack the pXO2 plasmid and retain pXO1, preserving the ability to secrete toxin [3,5,6]. Sterne-like strains exhibit higher levels of attenuation than Pasteur-like *B. anthracis* and have become the primary animal vaccine the world over. In 1994 and 2003, *B. cereus* carrying various pXO1 and pXO2 homologues caused isolated incidences of inhalational illness in the U.S. [7,8]. In 2001, an anthrax fatality event among apparently healthy primates was observed in the forests of Côte d’Ivoire [9]. This was unusual because anthrax outbreaks typically occur sporadically in ungulates due to their herbivorous grazing lifestyles. Investigations identified a novel *B. cereus* as the causative agent of these outbreaks. This *B. cereus* strain shared many characteristics with *B. anthracis* and contained two plasmids, one encoding anthrax toxins and a hyaluronic acid capsule on pCIXO1 (a pXO1 homologue carrying additional capsule genes) and the other encoding the poly-g-D-glutamic acid capsule on pCIXO2 (a pXO2 homologue)[10,11]. After finding this pathogen caused anthrax over wide swaths of forest in West and Central Africa, the realization dawned that this was a pathogen that had emerged recently and had been causing anthrax for years if not decades unbeknownst to scientists [12]. Archival samples were screened and the pathogen, now known as *Bacillus cereus* biovar *anthracis* (Bcbva), was isolated from bone samples dating back to 1996. As previously noted, *B. anthracis* cured of pXO2 becomes highly attenuated. When the pXO2 homologue is cured from Bcbva, there is only a slight decrease in virulence [13]. 

The findings meant that Bcbva had been causing anthrax in Africa for at least 5 years before a new organism became the suspect. Archival isolates provided a genetic snapshot of the emerging pathogen. It is still not clear where or when Bcbva exactly emerged; however, the U.S. isolates are closely related to the *B. cereus* background and could be similar to intermediates that acquired plasmids in Africa. Multiple-locus variable number tandem repeat analysis (MLVA) genotyping and later single nucleotide polymorphism (SNP) typing showed Bcbva nesting into its own clade outside of the *B. anthracis* group while multilocus sequence typing (MLST) indicated the closes relatives were two other *B. cereus* group bacteria associated with the diseases *Bacillus thuringiensis* serovar konkukian strain 97–27 and *Bacillus cereus* strain E33L [10,11]. 

In this work, archival monkey bone samples from the endemic forest anthrax zone were analyzed for the presence of Bcbva. We were able to isolate Bcbva from several archival samples that had been in the process of anthropological analysis. Genetic characterization and analysis of the bacterial strains and collection date of these bone samples mean we isolated the earliest known Bcbva by several years. The virulence plasmids and chromosomal markers of the strains were verified using multiplex PCR and the genomic material was sequenced to generate de novo assembled genomes. In silico MLVA typing and MLST were performed to characterize the strains. Sequence read alignment to CI revealed plasmid and chromosomal features that were conserved. Whole genome phylogenetic analysis was used to further characterize the relatedness and discriminatory power of Bcbva phylogenomic analysis. Over 100 Bcbva raw read files were extracted and analyzed with the addition of our newly isolated ancestral Bcbva in the context of host species, geospatial location, and temporal details. The capacity for whole genome SNP analysis to differentiate closely related strains isolated from the same host animals was also investigated. 

## 2. Results

### 2.1. Isolation and Identification of Bcbva from Archival Bone Samples

**Figure 1:**
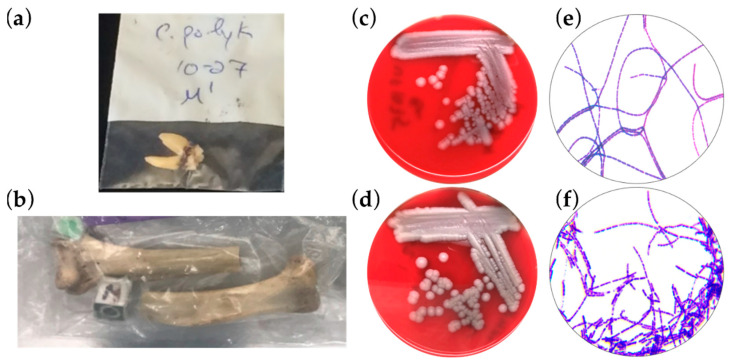
Isolation of Bcbva from archival bone samples from Taï National Park, Côte d’Ivoire: (**a**) The tooth of a king colobus (*Co. polykomos*) that died in 2010 from which Bcbva was isolated and positively identified. (**b**) The left humerus of a sooty mangabey (*Ce. atys*) that died of anthrax between May 1993 and August 1994 from which Bcbva was isolated and positively identified. (**c**) Anthrose-negative *B. anthracis* from Nigeria struck on sheep blood agar plate is very similar in appearance to Bcbva on the same media (**d**). (**e**) Gram’s stain of *B. anthracis* showing long Gram-positive chains. (**f**) Gram stain of Bcbva showing Gram-positive bacilli and chains.

Bones from five cercopithecoid taxa (*Cercocebus atys, Cercopithecus diana, Colobus polykomos, Pilliocolobus badius,* and *Procolobus verus*) were processed by bead-beating tissues or accessing marrow in long bones. Of 177 bone and tooth samples from 53 animals, Bcbva was found in four bones from three animals (Table 1) in addition to a previously described strain, UFBc0001 [14]. A tooth from one black and white colobus, one humerus from a different black and white colobus, and two samples from one sooty mangabey individual (mandible and humerus) from another, and the right humerus of a third yielded bacteria that were phenotypically similar to *B. anthracis* (Figure 1). The ethanol resistant spores grew vegetative bacteria that were positive to *lef*, *capB*, and genomic island 4 qPCR assays while negative for *B. anthracis*-specific markers [14]. 

### 2.2. In Silico Multilocus Sequence Typing, MLVA, and Genomic Analysis of Bcbva

The *B. cereus* MLST allele profile was obtained from our strains and the ST for all was determined as ST 935 as downloaded from pubMLST database [15,16] (Table 2). The ST of several Bcbva strains isolated from geographically distant locations in Africa also had ST 935. Using MLST, strains from infections in Côte d’Ivoire were identical to strains from the Democratic Republic of Congo nearly 1500 miles (2400 km) away. Partial profiling of Bcbva is possible with the MLVA-15 *B. anthracis* MLVA-typing scheme and it was found that all Bcbva matched the Bcbva CI strain alleles in the public *B. anthracis* MLVA database [17]. 

**Figure 2:**
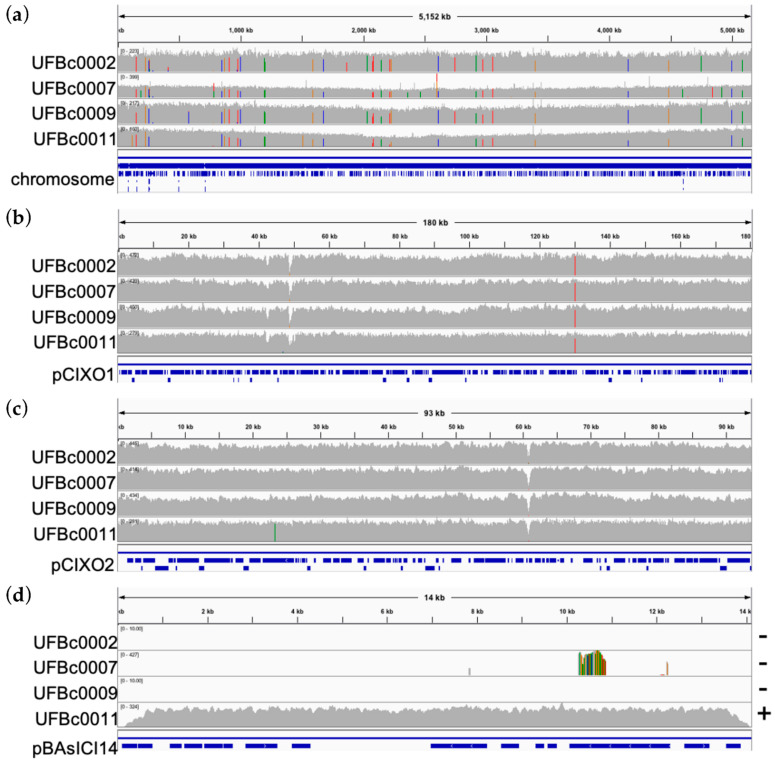
Read alignment to genomic features of Bcbva type strain CI: (**a**) Read alignment to the chromosome. (**b**) Read alignment to the pCIXO1 toxin-encoding virulence plasmid of strain CI. (**c**) Read alignment to the pCIXO2 capsule-encoding virulence plasmid of strain CI. (**d**) Read alignment to plasmid pBAslCI14. Gray is read coverage from BWA-MEM alignment to strain CI. Different colored vertical lines indicate SNP divergence of reads compared to Bcbva CI across the linearized gDNA segments.

Alignment of sequence reads (Figure 2a–d) to type strain CI have high levels of homology across the genomes. SNPs in the genomes compared to type strains CI are represented by colored bars in the gray coverage windows, while white dips in the gray coverage window indicate sequences present in CI that are not in our strains of interest. BWA alignment showed the presence of virulence plasmids pCIXO1 and pCIXO2 in UFBc0002, UFBc0007, UFBc0009, and UFBc0011 (Figure 2b,c). A single high-confidence SNP in pCIXO1 is shared among these strains compared to Bcbva CI (Figure 2b). Potential short sequence deletions in pCIXO2 of our strains compared to type strain CI and a SNP in pCIXO2 from UFBc0011 were observed (Figure 2c). Of these strains, only UFBc0011 was found to contain the plasmid pBAslCI14 (Figure 2d). The function of this plasmid is cryptic and is found sporadically in virulent Bcbva. Base pair mismatches are visible as different colored bars against the gray alignment histogram when aligned to Bcbva CI. Strain UFBc0011 is the most homologous to type strain CI and is also the closest in isolation date. Base pair mismatches between the earlier strains UFBc0002, UFBc0007, and UFBc0009 are SNPs representative of the ancestral Bcbva. 

### 2.3. Earliest Isolate Shows Similarity to Ancestral B. cereus 

**Figure 3:**
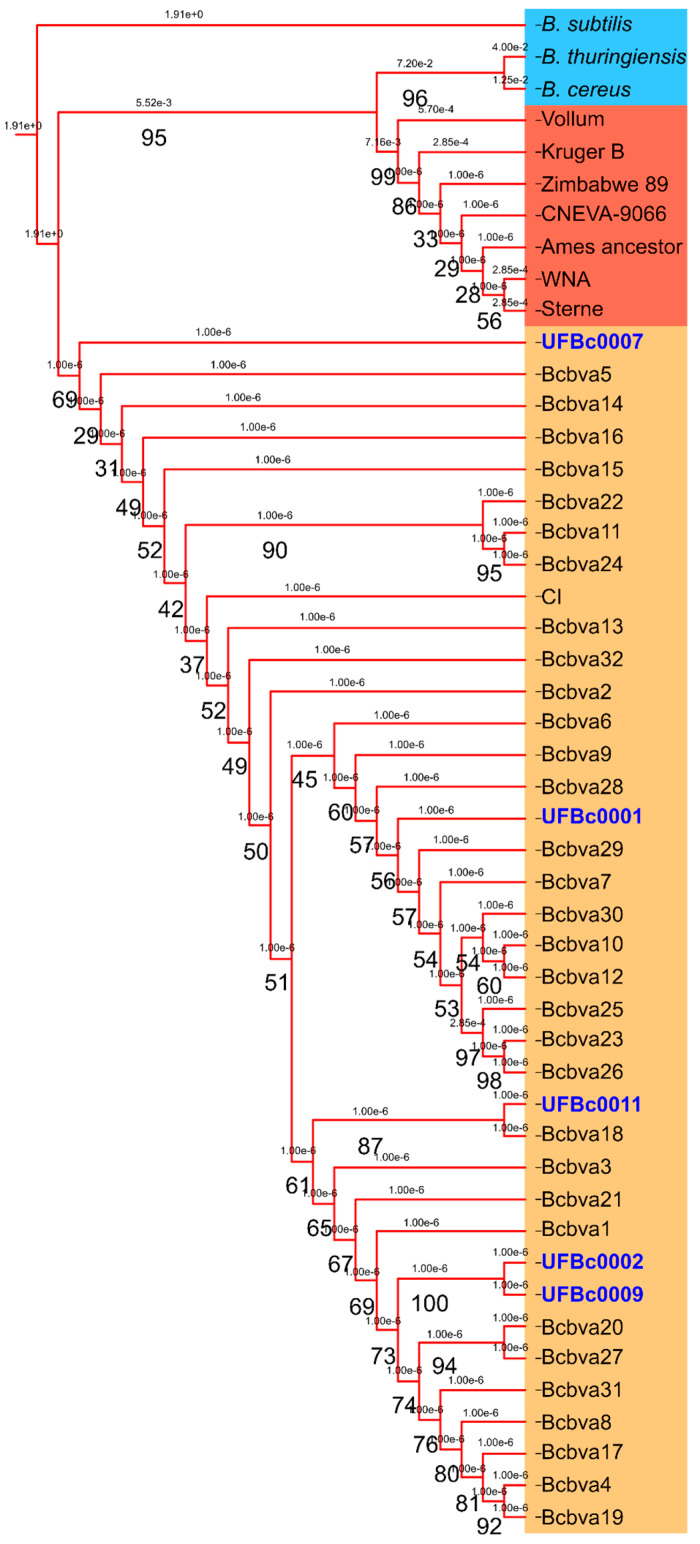
Maximum likelihood tree of chromosomal SNP alignments: Bcbva strains isolated in this study (blue font, orange background) were compared to other Bcbva sequences (orange background), *B. anthracis* type strains (red background), and other *B. cereus* group species, *Bacillus subtilis, B. cereus,* and *Bacillus thuringiensis* (blue background). UF Bc0007 isolated from an animal carcass from 1994 clustered closest to the *B. anthracis* and *B. cereus* outgroup, indicating it is the most ancestral Bcbva isolated to date.

A selection of chromosomal sequences from *B. anthracis* type strains, non-anthrax-causing *Bacillus* strains, and 33 Bcbva genomes (type strain CI and 32 other strains from Taï National Forest, Côte d’Ivoire) were analyzed with the PhaME module from Los Alamos National Labs (Figure 3). The non-anthrax-causing *Bacillus* spp. clustered, with *B. cereus* showing close and significant linkages to the anthrax-causing type strains. As expected, anthrax-causing Bcbva are present as an outgroup most closely related to *B. cereus.* PhaME analysis placed one of our earliest bone isolates from between May 1993 and August 1994, UFBc0007, as the Bcbva most closely related to the nonanthrax *B. cereus* and *B. anthracis*. This isolate was from a king colobus (*Colobus polykomos*) monkey that died in Taï National Forest. Other strains from this period, UFBc0002 and UFBc0009, were from a different animal (*Cercocebus atys*; sooty mangabey), also from Taï National Park, and were phylogenetically distinct from UFBc0007 yet still had high nucleotide homology. 

### 2.4. Whole Genome SNP Trees Reveal Spatial and Temporal Patterns of Infection

**Figure 4:**
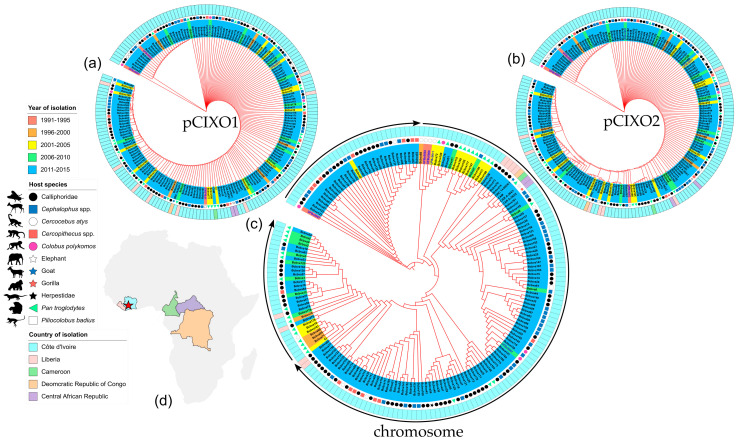
Phylogenetic tree showing relatedness of all 189 available Bcbva genome sequences (inclusive of 5 isolated in the lab). Different color labels under strain names indicate year of isolation. The symbols outside of the strain label ring indicate the host species pathogen was isolated from. The outer color rings indicate the country of origin. (**a**) The absence of genetic diversity among the toxin-encoding plasmid pCIXO1 is apparent in the low levels of branching between samples. (**b**) Amongst the Bcbva, capsule-encoding plasmid pCIXO2 shows more diversity than the pCIXO1 plasmid. (**c**) Maximum likelihood clustering using chromosomal SNPs as an input was able to cluster strains temporally and spatially. Black arrows on the outside of the circular tree indicate three temporal clusters of genetically related strains. (**d**) This inset map shows the location of the indicated countries whose color corresponds to the legend and the outer color rings of the circular phylogenies. The star on the map indicates the position of Taï National Park in Côte d’Ivoire.

Virulence plasmid sequences and chromosomal sequences were extracted from all publicly available sequenced Bcbva and sequence read archives and used to generate comprehensive phylogenetic trees. The total number of strains was 188 and included the 5 new strains isolated from the archival bone samples in our lab. Year of infection, host species and country of origin data were tabulated to provide insight to the phylogenetic relationships. Many of the strains were isolated from blow flies (Calliphoridae) and sequenced in a previous work [12]. Additional Bcbva strains were from animal anthrax mortalities across Africa and host species, including from an elephant, goat, and gorilla. The trees were rooted with the earliest isolate, UFBc0007. What is immediately apparent is the homogeneity of the toxin-encoding plasmids pCIXO1 at the sequence level (Figure 4a). A majority of the pCIXO1 sequences were identical with minimal spatial (outer ring colors) or temporal clustering (samples tag color) apparent. Sequences from the Central African Republic were located next to each other (purple area of outer ring). Capsule-encoding plasmid pCIXO2 showed slightly higher sequence variability (Figure 4b). Phylogenetic clustering from the pCIXO2 perspective was fairly conserved with that observed in pCIXO1 over the first half of the tree, after which variability among strains increased. Interestingly, pCIXO2 clustering found the Democratic Republic of the Congo strain (orange area) clustering with a subset of the Liberian isolates (pink). In both pCIXO1 and pCIXO2 trees, there was no pattern of host species observed (shape symbols at the end of the tag). However, when phylogenetic analysis using the chromosomal sequences of the Bcbva samples was carried out, distinct geographic, temporal, and epidemiological patterns emerged (Figure 4c). Temporally, three different lineages of strains were observed with the sample tags indicating year of infection in order from earliest to latest: red, orange, yellow, green, and blue. Analysis using the chromosome sequence allows for sample clustering by host species, potential artifacts of group mortality, and fly collection events. The geospatial clustering points towards Bcbva strains of a highly similar chromosomal sequence causing disease across a wide swathe of sub-Saharan Africa (outer ring color, specifically clustering of samples from Liberia, Cameroon, DRC and CAR).

### 2.5. Whole-Genome SNP Trees Allow for Accurate Case Clustering

**Figure 5:**
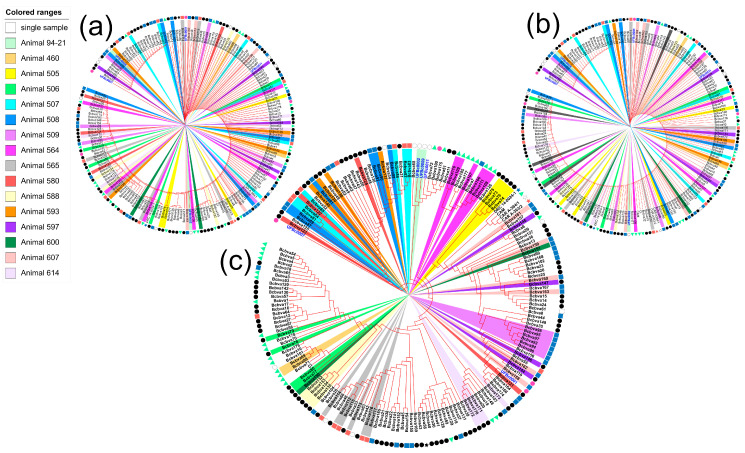
Analysis of between and within host clustering of Bcbva: The circular trees are the same as those presented in Figure 4. In several cases, multiple strains from a single animal were sequenced. The slices of each strain were colored in coordination with the animal they were isolated from. (**a**) Neither phylogenetic comparison of pCIXO1 sequences nor (**b**) pCIXO2 sequences was able to correctly group strains from the same animals. (**c**) The chromosomal SNPs allowed for clustering of many isolates from the same host animal. Symbols on the outer ring indicate host species.

Some samples in the tree were isolated from the same fatal anthrax infection, meaning multiple isolates were sequenced with high homology. We assessed whether plasmids or the chromosome could serve as a fine-scale epidemiological tool by observing the clustering of strains from the same animal (same colored pie slices) amongst samples from a single host (white slices) (Figure 5a–c). Like our finding with the temporal/spatial analysis, the low variability of the plasmids sequences resulted in limited clustering power as evidenced by the random color distribution of radial slices in the pCIXO1 and pCIXO2 trees. This was expected due to the highly clonal nature of the plasmids within Bcbva. The chromosomal sequences allowed a more robust clustering of samples isolated from the same host and can be seen with similar slice colors clustering together. While genomic variability among bacteria infecting a single host is a possibility, it cannot be ruled out that blow fly samples contained more than one fly or that that a fly had fed on numerous infected carcasses. Another possibility is that a fly carrying multiple diverse strains infected a single mammalian host. In fact, the clustering of blow fly (Calliphoridae) isolates (black circles in the host species ring) could reflect the fly feeding on the nearby carcasses prior to collection in the fly traps, highlighting the role of blow flies as a sentinel for Bcbva anthrax in the forest. 

### 2.6. Spatial Analysis of Genetic Similarity 

**Figure 6:**
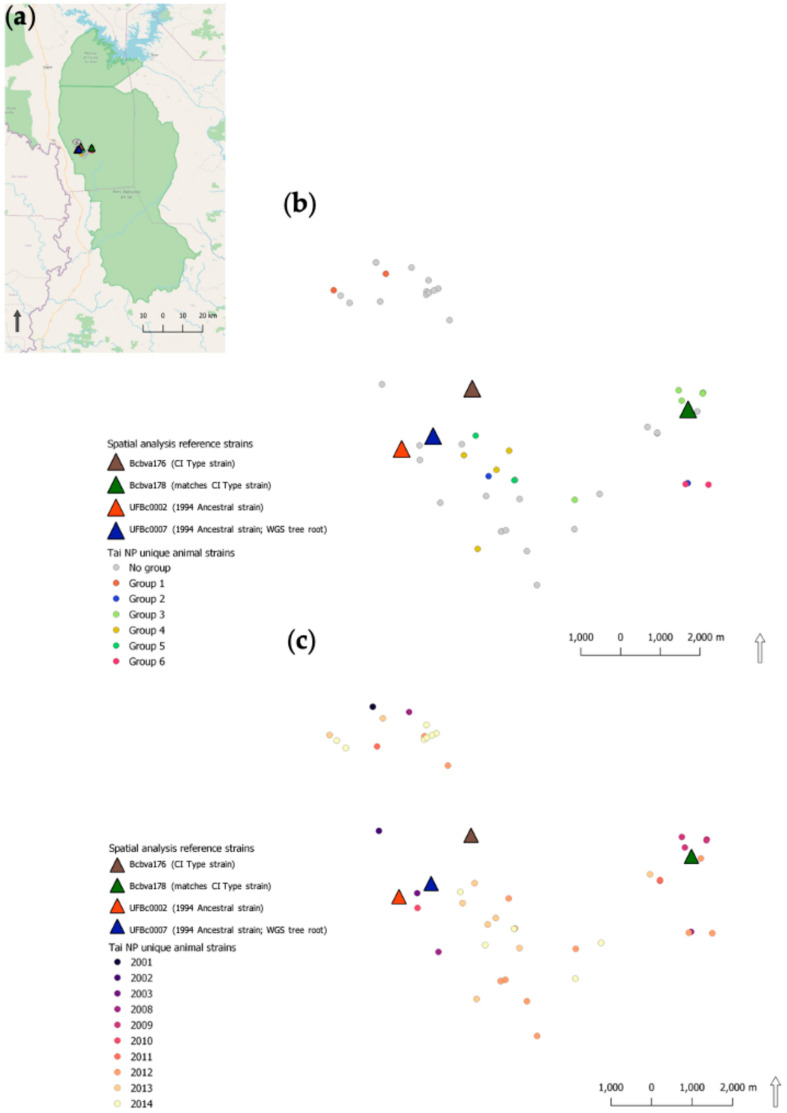
Phylogeographic analysis of Bcbva within Taï National Park, Côte d’Ivoire: (**a**) Political boundary map of Taï National Park (green area) in the west of Côte d’Ivoire with mortality events indicated by circles and reference strains from this work indicated by triangles. (**b**) Dummy genotypes of Bcbva strains based on RAxML phylogenies. (**c**) Bcvba isolate locations by year of collection. Triangles indicate the location of isolation of Bcbva-type strains in all three maps.

**Figure 7:**
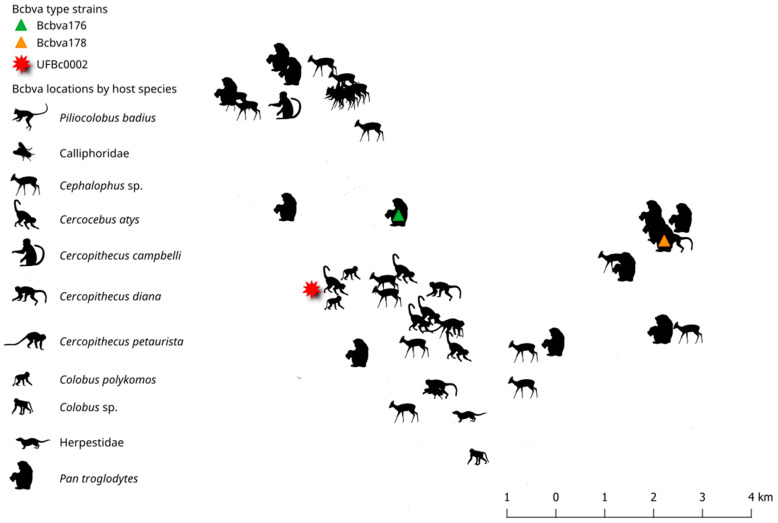
The geographic distribution of isolates by host species within Taï National Park, Côte d’Ivoire.

The distribution of isolates that could be mapped are displayed by genetic similarity and year of isolation (Figure 6). The average nearest neighbor index (ANNI) revealed a global spatial clustering of carcass locations (within a minimum bounding polygon representing the study extent within the park; ANNI: 0.6026, observed mean distance: 363 m, expected mean distance: 603 m, Z-score: −5.534). Within this overall carcass clustering, we identified several interesting patterns of Bcbva diversity (despite relatively high similarity across strains). We categorically assigned strains with near-identical genomes to dummy genotype groups 1–6 (colored dots) determined from hierarchical clustering of strains using genetic distances extracted out of the RAxML phylogenies. Like genotypes were found relatively close together. Generally, years were not specifically tied to genotype group. For example, Group 3 had several isolates in 2009, then again in 2012. Strains in later years were widespread across the study area, suggesting that year of collection did not account for spatial patterns. A lack of temporal dependence in the spatial clustering of like genotypes alludes to undetected transmission or animal exposures to dormant spore pools. Bcbva cases by host are mapped are in Figure 7. 

**Figure 8:**
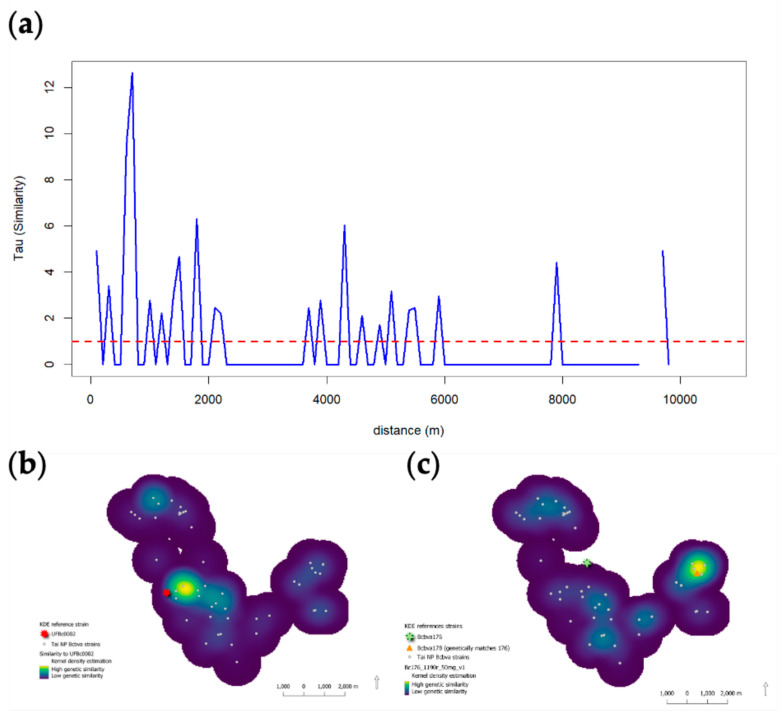
Phylogeographic analysis of Bcbva within Taï National Park, Côte d’Ivoire: (**a**) Tau statistic indicating highest degree of similarity to UFBc0002 ancestral strain in strains closest in geographic space. (**b**) Kernel density estimation (KDE) of genetic similarity to the UFBc0002 ancestral strain or (**c**) Bcbva178 (CI type like) were more like strains nearby; brighter colors indicated similarity is greatest in close proximity. Together, Tau and KDE indicate local patterns of transmission.

The Tau statistic was used to measure genetic similarity between strains and UFBc002 and the geographic location of host carcasses. Tau indicated relatively high similarity across Bcbva strains, as indicated by values above 1 (or above the red line in Figure 8a). However, the greatest Tau values were identified at short distances of less than 1000 m. Kernel density estimation (KDE) was used to map local patterns of similarity across space as a continuous surface examining similarity to each UFBc002 and archival strain Bcbva178 (CI Type like). Each KDE surface revealed concentrations of genetic similarity in close proximity to the reference strain (Figure 8b,c). These data suggest localized transmission. 

**Figure 9:**
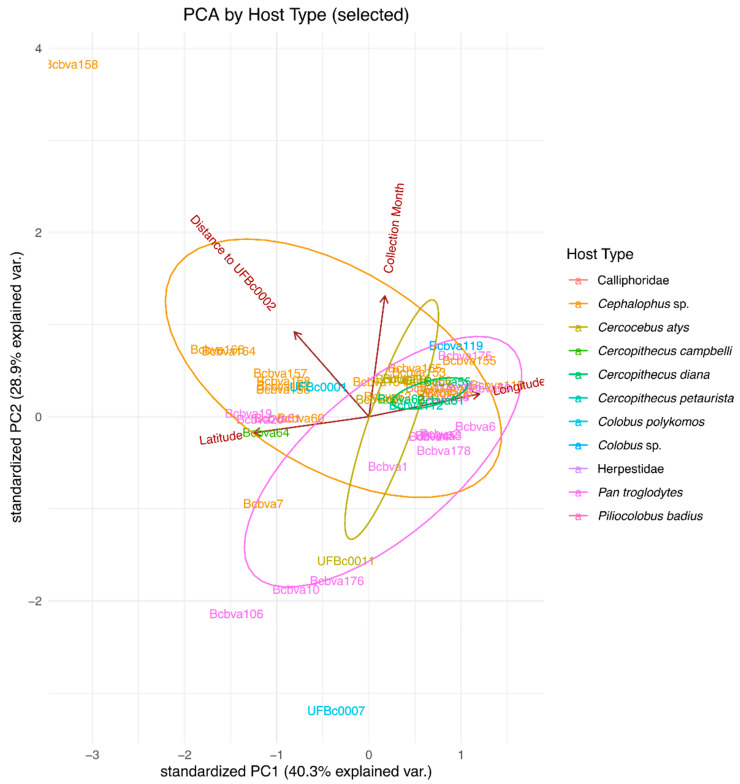
Principal component analysis of available Bcbva infection data. PCA analysis of strains was carried out using available sample metadata with labels colored by host species. Ellipses indicate PCA confidence intervals among the different groups. Ellipses in order of size from smallest to largest are *Cercop. diana* (Diana monkey)*, Ceroc. atys* (sooty mangabey), *Cephalophus* sp. (duiker), and *P. troglodytes* (chimpanzee) and indicate increasing variance in strains found among the host groups. Host icons in the legend match map symbols in Figure 5, Figure 7 and Figure 9.

Using principal component analysis (PCA), the proportion of variance explained by the components were 0.4026, 0.2892, 0.1745, and 0.1337, respectively. In total, the first two components explained 40.26% + 28.92% = 69.18% of variability. The first two components are visually summarized and presented in Figure 9 featuring color coding based on the host type and ellipses indicating groupings. Potential clustering was observed among strains isolated from *Cephalophus* sp., *Pan troglodytes*, *Cercocebus atys*, and *Cercopithecus diana*. Overlapping of host type groups indicate where the variability among the most important components is similar between the clusters. For *Pan troglodytes*, the largest variability was observed with geographic coordinates while more genetic diversity in terms of distance was identified for *Cephalophus* spp. The most compact cluster in terms of geographic variability was observed for *Cercopithecus diana.* Strains isolated from *Cercop. diana* were completely enclosed at the intersection of ellipses generated from the *Cephalophus* sp. isolated strains and the *P. troglodytes* isolated. The overlap in strains from these three groups could indicate a shared niche (e.g., food source) or interaction allowing for transmission of Bcbva between them. *Pan troglodytes* are known to hunt and eat *Cercopithecus* sp. and *Cerocebus* sp. monkeys in Taï National Park [18]. *Pan troglodytes* in Taï National Park were not commonly observed eating duikers (*Cephalophus*). Occasionally, juvenile chimpanzees would harass and kill young duikers, though not consume them. These close contacts could potentially transmit Bcbva from animal to animal or contaminate areas nearby, thus perpetuating infection. 

## 3. Discussion and Conclusions

Primate bones were exported from Côte’d Ivoire, Africa for anthropological studies [19]. These bones were from animal deaths occurring as early as from 1994 until 2010. In the intervening years after export from Africa to the United States, a novel pathogen causing anthrax disease was discovered in Western and Central Africa. The pathogen was genetically *Bacillus cereus*, normally a saprophyte occasionally causing foodborne illness, which had acquired homologues of the *B. anthracis* virulence plasmids. The anthrax toxin-secreting (pCIXO1) and capsule-producing (pCIXO2) bacterium was termed *Bacillus cereus* biovar *anthracis*—Bcbva [12,20]. A warning that these bones could have been from monkeys infected with Bcbva was sent to the sample importers (DJD, JK, SM). After relocating the samples to a select agent-certified BSL3 lab, bacteria were isolated from the archival bone samples and verified as Bcbva using an inhouse quadruplex qPCR. The quadruplex qPCR differentiates *B. anthracis* from Bcbva using species-specific chromosomal markers and each of the virulence plasmids [14]. Five strains of Bcbva were isolated from the remains of four animals: two *Co. polykomos* and two *Ce. atys* monkeys. The animals were found dead during observational studies in the Taï National Park in Côte’d Ivoire [21]. Three out of four of these animals were considered adults, the fourth a 1–2-year-old juvenile. Bacterial spores were isolated from decomposed tissue surrounding teeth and mandibles and from marrow inside long bones (humerus). The marrow of bones is an ideal environment for long-term survival of bacterial spores. On blood agar, the Bcbva isolates were nonhemolytic with classic colony *B. anthracis* morphology and indistinguishable from anthrose-negative *B. anthracis* that cause anthrax in nearby areas of West Africa. Gram’s stain showed minor differences between the two, mainly slight differences in the chaining of the vegetative organism. In the absence of molecular tests, a discerning eye would have considerable difficulty differentiating *B. anthracis* from Bcbva. 

In silico MLST profiling with the classical seven marker MLST system was carried out. Classical MLST profiling assigned our Bcbva strains to ST 935 along with other strains of Bcbva but was unable to differentiate them from one another. Integrative genome alignment of reads with the Bcbva-type strain CI revealed diagnostic SNPs that could be used to identify early emergence Bcbva and confirmed presence of toxin plasmid pCIXO1 and capsule plasmid pCIXO2. A previously described plasmid of unknown function that has variable presence in Bcbva was found in one of our strains, UFBc0011. The number of SNPs across the UFBc0002, UFBc0007, UFBc009, and UFBc00011 genomes compared to Bcbva CI were 2, 0, 0, and 12, respectively. There was a single SNP across pCIXO1 of all Bcbva strains sequenced in this work and no SNPs observed on pCIXO2, indicting a relatively recent ancestral acquisition of the toxin- and capsule-encoding plasmids by the type strain CI. Due to high levels of genetic homogeneity, we used chromosomal SNP clustering to analyze the relatedness of these strains. SNP analysis placed strain UFBc0007, isolated from an August 1994 *C. polykomos* (colobus monkey) carcass, as closer to the ancestral branch of the Bcbva group than any other Bcbva strains, providing an earlier genetic snapshot of pathogen emergence than previously available. 

Plasmid SNP analysis found diversity in pCIXO1 and pCIXO2 was mostly conserved but did not reveal temporal or spatial patterns, again likely due to recent potentially coincident acquisition of both virulence plasmids in the evolutionary history of *B. cereus*. Chromosomal SNP trees revealed temporal and broader spatial patterns of relatedness, connecting cross continent outbreaks of genetically similar Bcbva. For accurate molecular epidemiological trace-back, chromosomal SNP comparison is recommended due to the high level of genetic homogeneity among virulence plasmids in Bcbva. SNP analysis of the virulence plasmids was unable to group strains isolated from the same animals, while chromosomal analysis grouped strains from the same individual primate closely. These primate strains were interspersed with strains isolated from flies (Calliphoridae) collected in traps, supporting the hypothesis that flies are active spreaders of Bcbva in the forest [12,22,23,24]. 

Location of anthrax cases analyzed here were not linked temporally (by year). However, there was a strong genetic link between Bcbva strains and the location of anthrax cases. Strain similarity was highest in cases geographically closest to the reference strain selected and was consistent regardless of which reference strain, again supporting local transmission (e.g., flies). The disconnect between year of case and genetic relatedness of strains is likely an artifact of the dormant nature of spores, prognosticating the important role of the environmental pathogen sink. The intensive sampling and genomic data from this set of cases over a limited geographic range could allow future approximations of localized infectious zones (LIZs [25,26]) in forested anthrax ecosystems such as Taï National Park. Furthermore, PCA allowed us to determine variance in strains that supported the spatio-genetic signals. The variance among strains within a host type was inconsistent (not dependent on the same variables), highlighting the influence different animal behaviors may have on transmitting disease in the unique ecology of rainforest anthrax. 

Our study shows that Bcbva spores can survive decades and remain infectious in animal material. The earliest strain from this study was isolated in 1994 and had plasmids almost identical to those from Bcbva type strain CI isolated in 2002. This slow rate of genetic change is expected from a recently emergent pathogen with a partially dormant lifestyle. Future work focusing on the differences in ecology of Bcbva-caused anthrax and classical *B. anthracis* anthrax is warranted given how little is known and their overlapping geographies in Western and Central Africa. The recent and, for at least a decade, unknown emergence of Bcbva is a continued reminder that slowly evolving pathogens still harbor many surprises that can go undetected for years, necessitating continued biosurveillance efforts. 

## 4. Materials and Methods

### 4.1. Isolation and Verification of Bcbva from Bones and Teeth

Bones and teeth from monkey carcasses collected from Taï National Park in Côte d’Ivoire between 1994 to 2010 were originally transported to the USA for anthropological studies [19]. Skeletal material from Taï Forest was transported under CITES export permits 2352, 000704, 000705, 001809. Long bones, bone fragments, teeth, and mandibles with teeth from several monkey species were catalogued and tested one-by-one for presence of viable bacteria. Some of these materials, like the teeth, still had tissue remnants attached and were bead-beat directly to isolate bacteria as previously described [27]. Other long bone samples were sawed in half to access the marrow. Dried marrow was subjected to bead-beating for isolation of bacteria. Nonhemolytic colonies on sheep blood agar (SBA) were qPCR verified using the Ba-1, GI4, *lef*, and *capB* assays, as previously described [27]. Multiplex qPCR developed by the lab was used to confirm genotypes prior to genome sequencing [14]. 

### 4.2. Genome Sequencing of Bcbva Performed Using the Illumina MiSeq

Genomic DNA (gDNA) was extracted from minimally passaged colonies grown on SBA and isolated using the QIAGEN DNeasy UltraClean Microbial Kit (Hilden, Germany), filter-sterilized through a 0.22 mm spin filter, and sterility verified according to entity-specific procedures and CDC guidance. Libraries were produced with the NEBNext Ultra II Library Prep kit (NEB, Ipswich, MA, USA), as described previously [28]. Briefly, 700–800 bp inserts were size-selected using magnetic beads and index barcode primers were added to fragments with minimal PCR cycles. Excess primers were removed in another round of magnetic bead size selection. The libraries were sequenced with the MiSeq next-generation sequencing system (Illumina, San Diego, CA, USA) at the UF Emerging Pathogens Institute using paired-end v3 chemistry. Read quality was visualized with FastQC and trimmed to remove poor quality bases (<Q30) and any residual adaptors with Trimmomatic [29], and SPAdes [30] was used for de novo assembly of genomes. Assembled genome quality was assessed with QUAST [31] and analyzed using CLC Sequence Viewer 7.5.0 (QIAGEN). Reference sequences were obtained from GenBank. BioSample accession numbers of the Illumina read files in the NCBI Sequence Read Archive for UFBc0002, UFBc0007, UFBc0009, and UFBc0011 are SAMN28772829, SAMN28772830, SAMN28772831, SAMN28772832, respectively, and have been deposited in BioProject PRJNA843952. Reviewer link https://dataview.ncbi.nlm.nih.gov/object/PRJNA843952?reviewer=o4h3fhnt76cgm5idksc33a64tc accessed on 16 August 2023. UFBc0001 was previously described and deposited in Bioproject PRJNA681734 as BioSample SAMN15804163 [14]. UFBc0001 was isolated from the same bone collection and was included in the analyses presented here.

### 4.3. Sequence Processing and Bioinformatics Analysis

BWA-MEM [32] was used to align the short Illumina reads to Bcbva-type strain CI and its plasmids. Alignments were viewed with Integrative Genomics Viewer version 2.5 (IGV; Broad Institute [33]). Assembled genomes were used for in silico MLST and MLVA typing to compare strains in the *B. cereus* MLST database and the *B. anthracis* MLVA database, respectively. The profiles were present in each but were unable to differentiate strains from different years or geographic locations. Chromosomal SNP mapping was used to identify genetic relatedness of early Bcbva to ancestral *B. cereus* and other *Bacillus*-type strains. To do so, whole-genome SNP alignments were created with the PhAME software package developed at Los Alamos National Labs [34,35], and phylogenetic trees were generated with RAxML [36] within PhaME and visualized with iTOL [37]. Raw sequence fastq files for Bcbva from [12] were obtained from the SRA database and assembled as described above. Host species and country details were compiled from the previously published works and combined with our newly sequenced strains for visualization with phylogenetic trees. The combined metadata is provided in Supplementary Table S1. Phylogenetic analysis of all Bcbva in the database found that plasmid sequence was too homogenous to allow for discrimination of strains and discriminatory power lay within chromosomal SNPs. Strains were MLST typed using the pubMLST database [16] according to the previously published *B. cereus* MLST schema [15]. Partial MLVA profiling was carried out using the MLVA finder python script [38]. Variant calling was accomplished by analyzing reads with FreeBayes [39] compared to Bcbva-type strain CI.

### 4.4. Exploratory Spatial Data Analysis of Bcbva Isolates from Taï National Park

For this study, we mapped isolates by date (year), host species, and location of carcass from published reports or case locations from animal remains tested here. This produced a subset of 54 mapped isolates for spatial analysis (Figure 5); sample size was reduced to 53 when comparing strains to UFbc0002 or 52 when comparing to CI type strains Bcbva176 and Bcbva178. We employed an exploratory spatial data analysis framework (ESDA [40]), where we first tested if the 54 case locations were clustered in space using the average nearest neighbor index (ANNI). To describe the distribution of strains, we mapped species by host and year. To map hosts, we assigned a unique animal shape to each species in the database. SVGs were downloaded from Shutterstock (Shutterstock, Inc., New York, NY, USA) under license. Additionally, we categorically assigned strains with near-identical genomes to dummy genotype groups 1–6 (colored dots) determined using hierarchical clustering of strains using genetic distances extracted from the RAxML phylogenies. Strains not closely related to other strains (using this WGS SNP approach) were not assigned to a group (no group—gray dots). Next, we used the Tau statistic to test whether isolates genomes of isolates were more similar when strains were closer together in geographic space [41]. The Tau statistic was calculated using the IDSpatialStats package in R and plotted using the ggplot2 package. Here, we were most interested in determining if isolates found close together in geographic space were more similar genetically, assuming a distance decay hypothesis. 

Next, we calculated descriptive spatial statistics, spatial mean and standard distance, for isolate locations weighting each location by similarity to each UFBc0002 or Bcbva178 (the CI-type strain) following Blackburn et al. [42]. While ANNI and Tau are useful for measuring global spatial patterns within the dataset, we were also interested visualizing local spatial patterns of genetic similarity. Toward this, we employed kernel density estimation (KDE). For the spatial descriptive statistics, and the subsequent KDE, we took the inverse of the similarity metric to the strain of interest. Since the similarity metric was a genetic distance value with values closer to zero indicating higher similarity to that type of strain, inverse values were very high when more similar. We then used KDE to visualize the spatial distribution of strain similarity to the strain type of interest per 100 m^2^ per across the study period [42]. For this study, we used the kernel density estimation tool in the SAGE toolbox of Q-GIS version 3.18.3 using the quartic kernel function [43]:

(1) $$f\left(x\right)=\frac{1}{nh}\;\sum_{i=1}^{n}K\left(\frac{x-X_{i}}{h}\right)$$

where *h* is the bandwidth and x*-Xi* is the distance to each isolate *i.* Finally, *K* is the quadratic kernel function, defined as

(2) $$K\left(x\right)=\frac{3}{4}\left(1-x^{2}\right),\;\left|x\right|\leq 1$$

(3) $$K\left(x\right)=0,x>1$$

This function was employed to estimate similarity to the strain type of interest weighted by each isolate’s inverse similarity. We calculated bandwidth (kernel) using *h_opt_* that uses the sample size (number of mapped isolates) and the standard distance (from the point distribution) to estimate bandwidth [44]. Here, we calculated the average *h_opt_* for each of the strains and applied to both analyses. The resulting outputs were map surfaces showing the concentration of genetic similarity across the study area. For this paper, we illustrated KDE for similarity to UFBc0002 (the oldest strain in the database) and Bcbva178 (one of two CI-type strains and identical to Bcbva176). Outputs were mapped with 20 equal interval bins, with values in bright yellow representing areas of high similarity. 

### 4.5. Principal Component Analysis of Sample Metadata and Genetic Similarity 

Principal component analysis (PCA) was used to further investigate the data and identify potential separations by host type and animal identifiers based on geographical coordinates (longitude and latitude), sample collection date, and the genetic distance of all sample elements to the oldest sequence in the sample, coded as UFBc0002. The subset of analyzed sequences with geographic coordinates consisted of 51 sequences, which was consistent with the other subsample used for geographic visualization and analysis. In the summary figure, the host type and animal identifiers were solely used for color coding and were not considered as variables for the PCA. Ellipses presented in the figure visualization were drawn to emphasize the grouping based on the host type. The PCA was performed in R software for statistical computing using the prcomp() function. The visualization was generated using the ggbiplot() function from the ggplot2 package. The ellipses were produced with the default settings of the ggbiplot() function to identify potential clusters.

## Figures and Tables

**Table 1:** Details of host animals and bone samples leading to Bcbva isolation.

SEER Lab ID	OSU Bone ID	Host Species	Age Estimate	Samples	Associated Pure Culture
38	10–27	*Colobus polykomos*	4–6 years; adult male	Tooth, M1	UFBc0001
78	94–21	*Cercocebus atys*	7–10 years; adult male	Left humerus	UFBc0002
102	94–21			Mandible scrapings and associated tooth	UFBc0009
83	94–31	*Colobus polykomos*	4–6 years; adult female	Right humerus	UFBc0007
174	23–10	*Cercocebus atys*	1–2 years; juvenile	Mandible fragment	UFBc0011

**Table 2:** Multilocus sequence typing (MLST) of relevant Bcbva strains and epidemiologic details.

	Locus							ST	Country	Year	Host Species
Strain	*glp*	*gmk*	*ilv*	*pta*	*pur*	*pyc*	*tpi*				
CI	34	1	83	1	18	29	5	935	Côte d’Ivoire	2002	*Pan troglodytes*
UFBc0001	34	1	83	1	18	29	5	935	Côte d’Ivoire	2010	*Colobus polykomos*
UFBc0002 *	34	1	83	1	18	29	5	935	Côte d’Ivoire	1993–94	*Cerocebus atys*
UFBc0007 *	34	1	83	1	18	29	5	935	Côte d’Ivoire	1993–94	*Colobus polykomos*
UFBc0009 *	34	1	83	1	18	29	5	935	Côte d’Ivoire	1993–94	*Cerocebus atys*
UFBc0011 *	34	1	83	1	18	29	5	935	Côte d’Ivoire	2003	*Cerocebus atys*
DRC-14-0024-1	34	1	83	1	18	29	5	935	DRC	2012	goat
CAR-A364-1	34	1	83	1	18	29	5	935	CAR	2013	*Gorilla gorilla*
CAR-A363-2	34	1	83	1	18	29	5	935	CAR	2012	Elephant
CAM	34	1	83	1	18	29	5	935	CAM	2014	*Pan troglodytes*
Bcbva69	34	1	83	1	18	29	5	935	Côte d’Ivoire	1996	*Pan troglodytes*
Bcbva172	34	1	83	1	18	29	5	935	Côte d’Ivoire	2014	Calliphoridae
Bcbva73	34	1	83	1	18	29	5	935	Liberia	2013	Calliphoridae
Bcbva80	34	1	83	1	18	29	5	935	Liberia	2013	Calliphoridae

DRC: Democratic Republic of Congo; CAR: Central African Republic; CAM: Cameroon; * indicates strains whole genome sequenced in this study.

## Data Availability

Whole-genome sequence data have been uploaded to public NCBI databases under BioSample accession numbers of the Illumina read files in the NCBI Sequence Read Archive for UFBc0002, UFBc0007, UFBc0009, and UFBc0011 are SAMN28772829, SAMN28772830, SAMN28772831, SAMN28772832, respectively, and have been deposited in BioProject PRJNA843952. Reviewer link: https://dataview.ncbi.nlm.nih.gov/object/PRJNA843952?reviewer=o4h3fhnt76cgm5idksc33a64tc (accessed on 16 August 2023).

## Supplementary Materials

The following supporting information can be downloaded at: https://www.mdpi.com/article/10.3390/pathogens12081065/s1, Table S1: *Bacillus cereus* biovar *anthrax* strain metadata.
